# Determination of the Qualitative Composition of Biologically-Active Substances of Extracts of In Vitro Callus, Cell Suspension, and Root Cultures of the Medicinal Plant *Rhodiola rosea*

**DOI:** 10.3390/biom11030365

**Published:** 2021-02-27

**Authors:** Lyudmila Asyakina, Stanislav Sukhikh, Svetlana Ivanova, Alexander Prosekov, Elena Ulrikh, Evgeny Chupahin, Olga Babich

**Affiliations:** 1 Department of Bionanotechnology, Kemerovo State University, Krasnaya Street 6, 650043 Kemerovo, Russia; alk_kem@mail.ru (L.A.); stas-asp@mail.ru (S.S.); 2 Institute of Living Systems, Immanuel Kant Baltic Federal University, A. Nevskogo Street 14, 236016 Kaliningrad, Russia; chupakhinevgen@gmail.com (E.C.); olich.43@mail.ru (O.B.); 3 Natural Nutraceutical Biotesting Laboratory, Kemerovo State University, Krasnaya Street 6, 650043 Kemerovo, Russia; 4 Department of General Mathematics and Informatics, Kemerovo State University, Krasnaya Street, 6, 650043 Kemerovo, Russia; 5 Laboratory of Biocatalysis, Kemerovo State University, Krasnaya Street 6, 650043 Kemerovo, Russia; a.prosekov@inbox.ru; 6 Kuzbass State Agricultural Academy, Markovtseva Street, 5, 650056 Kemerovo, Russia; elen.ulrich@mail.ru

**Keywords:** in vitro callus, cell suspension, root cultures, *Rhodiola rosea*, biologically-active substances, HPLC, ^1^H NMR spectra

## Abstract

The results of the qualitative composition analysis of the dried biomass extracts of in vitro callus, cell suspension, and root cultures show that the main biologically active substances (BAS) in the medicinal plant, *Rhodiola rosea*, are 6-C-(1-(4-hydroxyphenyl)ethyl)aromadendrin (25 mg, yield 0.21%), 2-(3,7-dihydroxy-2-(2-hydroxypropan-2-yl)-2,3-dihydrobenzofuran-5-yl)-6,7-dihydroxychroman-4-one (23 mg, yield 0.2%), 2-(3,4-dimethoxyphenyl)-5,7-dimethoxychroman-4-one (175 mg, yield 1.5%), 5,7-dihydroxy-2-(4-hydroxy-3-(2-(4-hydroxyphenyl)-4-oxo-4H-chromen-6-yl)phenyl)-4H-chromen-4-one (45 mg, yield 0.5%), 5,6,7,8-tetrahydroxy-4-methoxyflavone (0.35 mg, 0.5%). BAS from the dried biomass extracts of in vitro callus, cell suspension, and root cultures of *Rhodiola rosea* will be used for the production of pharmaceuticals and dietary supplements with antitumor, antimicrobial, and antioxidant effects.

## 1. Introduction

Since the appearance of in vitro technology, the ability of plant cell, tissue, and organ cultures to produce and store many valuable compounds has been recognized. Today’s strong and growing demand for natural and renewable products has shifted its focus to in vitro cell cultures as potential phytochemical factories [1,2]. The advantage of in vitro production of plant metabolites is the capacity to better understand the biological characteristics of their biosynthetic activity and, ultimately, to increase their biosynthetic activity by regulating physical, chemical, nutritional, and genetic parameters. Medicinal compounds located in specialized morphological tissues or organs of natural plants can be obtained not only by induction of certain tissue cultures but also by using undifferentiated callus/cell cultures in the culture system [3].

Advances in plant cell culture technology have made it possible to manufacture a variety of drugs such as alkaloids, terpenoids, steroids, saponins, phenolic compounds, flavonoids, and amino acids. The production of plant metabolites by cell culture has many advantages, such as the opportunity to select genotypes with a higher yield of secondary metabolites, which can be continuously produced throughout the year in a controlled environment. Plant cell culture removes potential political boundaries or geographic barriers that can impede crop production, such as limiting natural rubber production in the tropics or limiting anthocyanin production in the tropics in high light-intensity climates [4,5].

Callus/cell suspension cultures have been the subject of various studies aimed at obtaining phytochemicals that not only have medicinal value but also contain other industrially important metabolites. The callus is a product of the proliferation of undifferentiated cells and can be obtained in vitro from various explants of the same plant species on a suitable nutrient medium. Callus obtained from explants producing a large number of metabolites can be transferred into a liquid medium with constant stirring to obtain a suspension culture. Zenk [6] has successfully created a variety of plant cell lines that can produce secondary compounds in high yield in cell suspension cultures. Solanine, isolated from the callus of Solanum, and methetin, isolated from the callus culture of cephalosporins, were successfully produced [7]. Some well-known cell culture methods that have been used for large-scale production of metabolites include the production of paclitaxel from the suspension culture of *Taxus chinensis* cells [8]; the production of taxol and related taxanes from various yew species; the production of berberine using a suspension culture of berberine cells; the production of vincristine and vinblastine from periwinkle [9,10]; taxane compounds obtained from the yew cell suspension culture [11].

Recently, growing hairy roots has been considered a sustainable strategy for the production of medically important plant metabolites, not only because root harvesting is harmful to plants in nature, but also because hairy roots are easy to grow in the absence of external hormones, lack of geophilicity, and strong root branching in a large number of cultures [12,13]. Furthermore, compared to natural roots, hairy roots produce secondary metabolites for a longer period [14,15]. Natural roots are not only limited in quantity but can also be used only at certain times of the year. For these reasons, the transition from growing natural plant organs to hairy roots is considered an attractive option for the production of many valuable natural secondary metabolites [16,17].

To produce hairy root cultures, plants are infected with *Agrobacterium rhizogenes*, which induce hairy root formation by transferring the transfer DNA (T-DNA) from the Ri plasmids into the plant genome. This ability of *Agrobacterium rhizogenes* has led to research into its use as a source for root drugs [18]. Important metabolites produced by hairy roots include serpentine in *Catharanthus roseus*, absintin in *Rauvolfia micrantha* [19], and ginkgolides in the hairy roots of *Ginkgo biloba* [20]. By optimizing the organic nutrient content of the bioreactor to increase its yield, ginsenosides can be produced in large quantities from the hairy roots of ginseng. Recent developments have shown that hairy-root-growing methods have evolved from small laboratories to large-scale industrial production. For example, the German company ROOTec uses hairy root cultures to produce camptothecin and podophyllotoxin. In a co-culture system between species, it was found that flax hairy roots increased podophyllotoxin production in cell suspension by 240%. Reportedly, secondary metabolites accumulated in aerial plants also accumulate in hairy roots, for example, artemisinin, which is believed to accumulate only in aerial parts of plants. *Artemisia annua* also accumulates in the hairy roots. The use of various concentrations of auxin and cytokinin and their combination may result in a higher level of forskolin in roots transformed with callus [21]. According to available data, picroside-1 production in Kulhua hairy root cultures is increasing [22].

This work aims to study the qualitative composition of biologically-active substances (BAS) of extracts of in vitro callus, cell suspension, and root cultures of the medicinal plant *Rhodiola rosea.*

## 2. Materials and Methods

### 2.1. Research Objects

Complexes of biologically active substances (BAS complexes) isolated from extracts of freeze-dried biomass of in vitro callus, cell suspension, and root cultures of the medicinal plant *Rhodiola rosea*, collected in the Kemerovo region (Siberia, Russia) in 2020, were the objects of this research. To obtain the biomass of in vitro callus, cell suspension, and root cultures, the seeds of *Rhodiola rosea* were pre-washed with a detergent (hydrogen peroxide 3%), then immersed for 1 min in a 75% ethanol solution, transferred to a laminar box, and sterilized for 15 min in a 20% sodium hypochlorite solution (5% active chlorine). After sterilization, the sterilizing substance was washed off; the seeds were washed for 20 min in distilled sterile water three times. Then, the explants were placed in a sterile 100 mL flask with 30 mL of Murashige–Skoog culture medium containing 3% sucrose and 0.7% agar-agar, without growth stimulants, and were illuminated by compact fluorescent lamps Economy 11W/865 11W E27 3U 6500K 6y CDL Philips 871150031502110 (Philips, Eindhoven, The Netherlands), while maintaining a temperature of 25 °C. Seedlings aged 1.5 months old were used for the transformation. Lyophilization of germinated biomass of in vitro callus, cell suspension, and root cultures was carried out using a Triad freeze-dryer by Labconco (Kansas City, MO, USA). Lyophilization conditions selected involved a vacuum 0.05 mbar and a cooler temperature of −80 °C. The extracts were obtained as follows: a portion of the studied biomass sample was weighed on an analytical balance (Oxaus PX85, New York, NY, USA) and transferred into a polyethylene Falcon tube, an organic solvent (ethanol) was added in an amount of 1:5 according to the experimental procedure, and the extraction process was carried out. The duration and temperature of the experiment varied up to 360 min and from 25 °C to boiling, respectively. Further, the filtration process was carried out, followed by centrifugation of the filtrate at a rotor speed of 3900 ± 100 rpm. The filtrate was centrifuged in a PE-6900 centrifuge (Ekros, Moscow, Russia) to remove suspended particles. The solvent was evaporated from the extract on an IKA RV 8 V rotary evaporator (IKA, Staufen, Germany), under reduced pressure from a 100 mL flask pre-weighed on a CAS CUW420H balance (CAS Corporation Ltd., Seoul, Korea). The flask was weighed, and the yield of the extract was determined.

### 2.2. Drying of the BAS Complex

Drying of the BAS complex isolated from extracts of lyophilized biomass of in vitro callus, cell suspension, and root cultures was also carried out by lyophilization. Lyophilization was performed using a Triad freeze-dryer by Labconco (Kansas City, MO, USA). Lyophilization conditions made it possible to optimize the temperature and drying time of the samples. The following constant conditions for lyophilization were selected: vacuum 0.05 mbar and temperature of the cooler −80 °C. The temperature regime and duration of the lyophilization process were individually selected for each sample. The residual solvent content was the controlled parameter [23].

### 2.3. Separation and Identification of Individual BASs

The isolated BAS complexes from the lyophilized biomass extracts of in vitro callus, cell suspension, and root cultures of *Rhodiola rosea* were additionally separated by preparative HPLC using a Shimadzu chromatograph (Shimadzu, Kyoto, Japan), with a flow rate of 10 mL/min, phase A–B gradient of 1–90% in 15 min, and phase A–0.1% trifluoroacetic acid and B–acetonitrile [24].

To identify the BAS of in vitro callus, cell suspension, and root cultures extracts of *Rhodiola rosea*, a mixed stock solution was prepared immediately before the experiment, containing 1 mg/mL of each biologically active substance in ethanol. To construct a calibration curve, standard solutions were prepared by sequential dilution of the mixed stock solution with ethanol to final concentrations from 0.1 to 100.0 μg/mL. The solutions were chromatographed and eluted. We used an H_2_O:MeCN eluent system with an acetonitrile gradient of 0–20% with a step of 2%; trifluoroacetic acid was used as a modifier, which was added in an amount of 0.1%. The content of each BAS was calculated based on the predetermined calibration curves between the peak regions and the concentrations of the standard solutions.

Each fraction was evaporated to dryness, weighed, the yield determined, and the structure of the compounds was identified by proton (^1^H) NMR spectrometry.

^1^H NMR spectra were obtained using a Bruker AVANCE NMR spectrometer (Bruker, Leipzig, Germany) with an operating frequency of 500 MHz and with CDCl_3_ (chloroform- *d*) used as a solvent for all compounds [25].

## 3. Results

**Figure 1:**
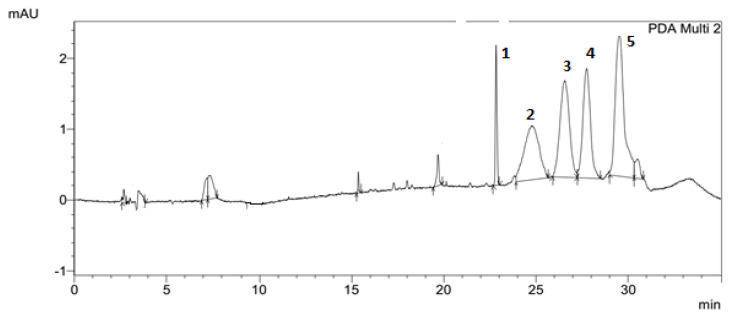
Results of preparative separation of flavonoid fractions from extracts of callus, cell suspension, and root cultures of *Rhodiola rosea*.

**Figure 2:**
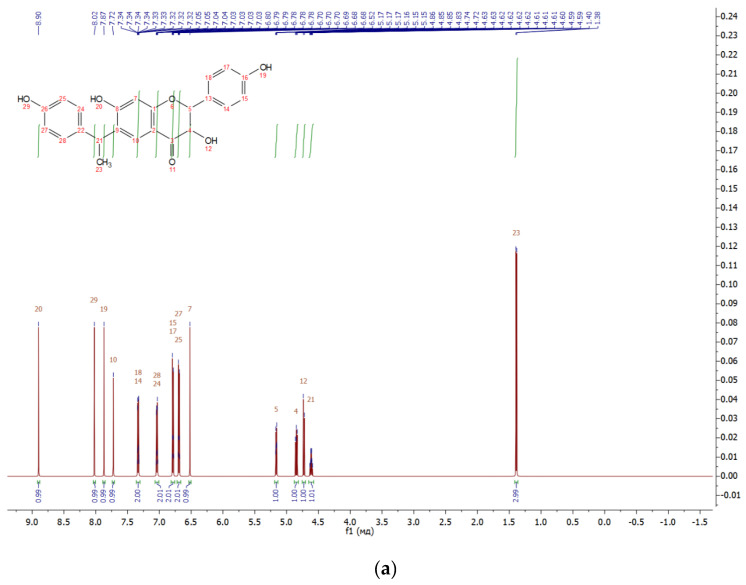

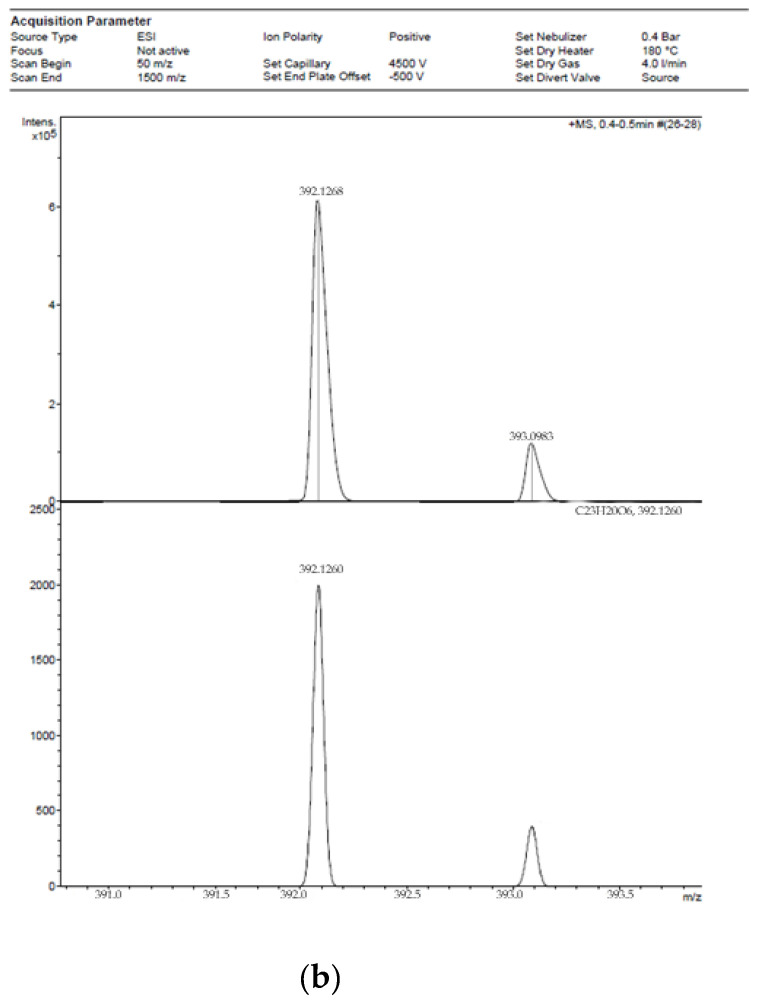
Proton (^1^H) NMR (**a**) and high-resolution mass spectrometry (HRMS) (**b**) spectrum of 6-C-(1-(4-hydroxyphenyl) ethyl) aromadendrin from extracts of callus, cell suspension, and root cultures of *Rhodiola rosea*.

**Figure 3:**
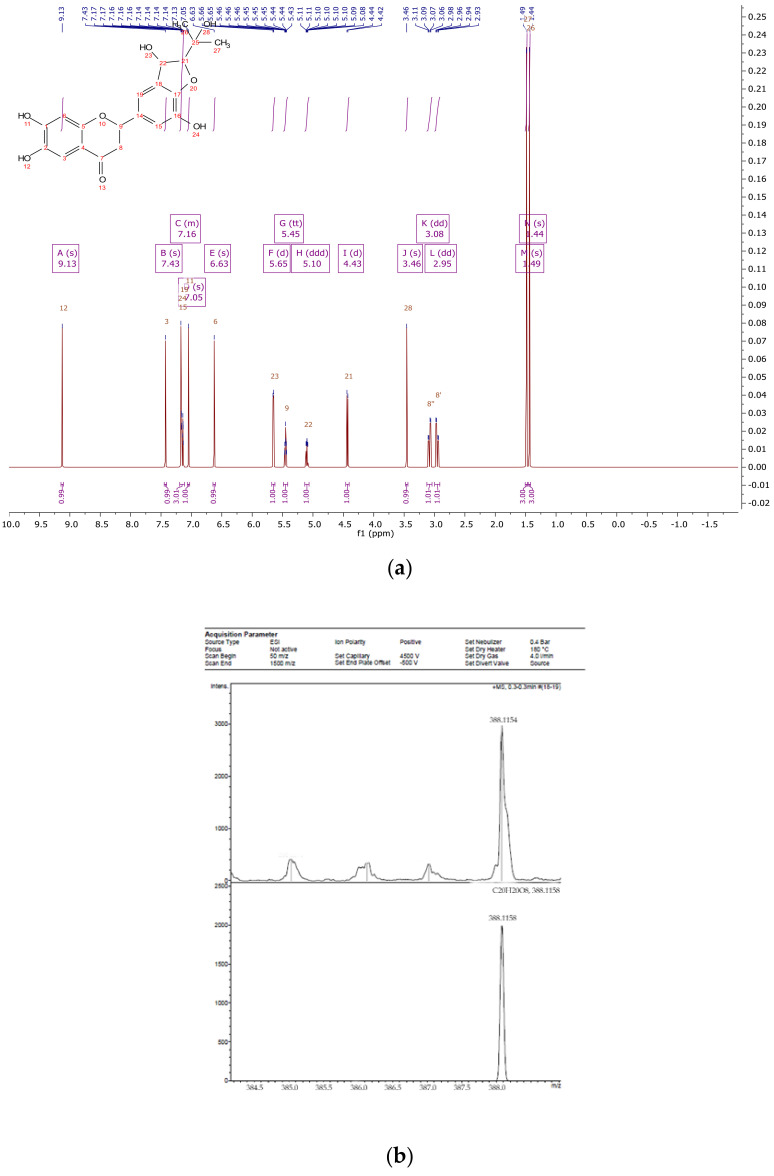
^1^H NMR (**a**) and HRMS (**b**) spectrum of 2-(3,7-dihydroxy-2-(2-hydroxypropan-2-yl)-2,3-dihydrobenzofuran-5-yl)-6,7-dihydroxychroman-4-one from extracts of callus, cell suspension, and root cultures of *Rhodiola rosea*.

**Figure 4:**
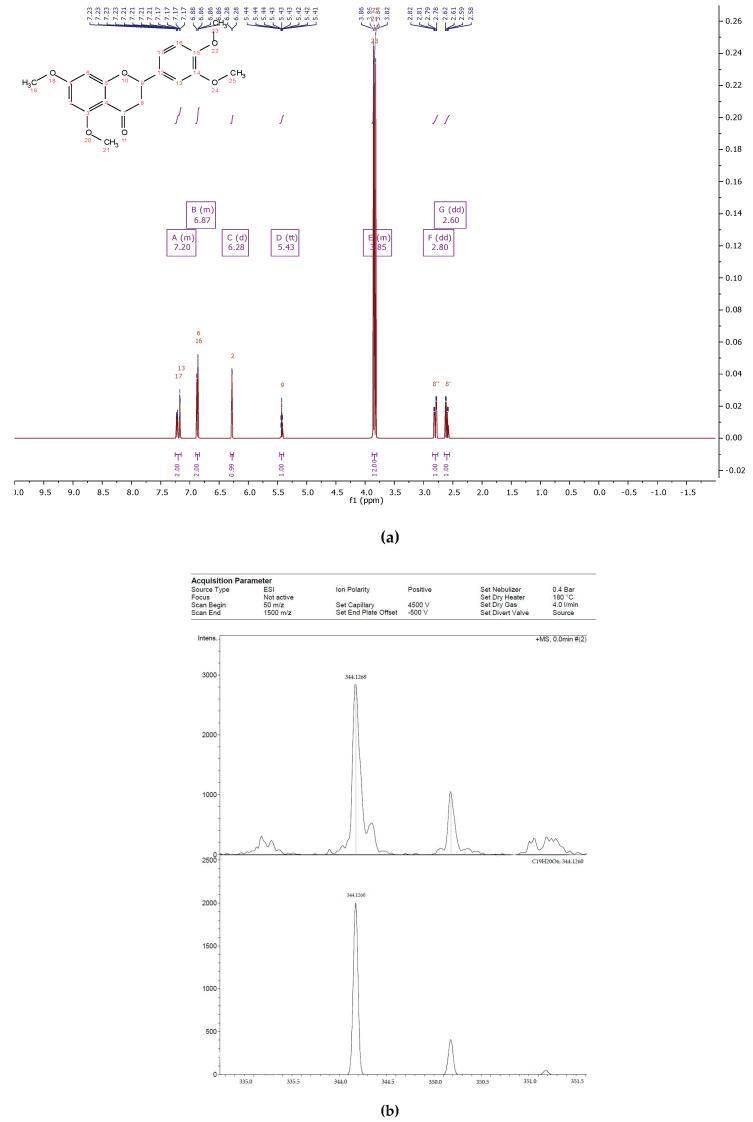
^1^H NMR (**a**) nd HRMS (**b**) spectrum of 2-(3,4-dimethoxyphenyl)-5,7-dimethoxychroman-4-one from extracts of callus, cell suspension, and root cultures of *Rhodiola rosea*.

**Figure 5:**
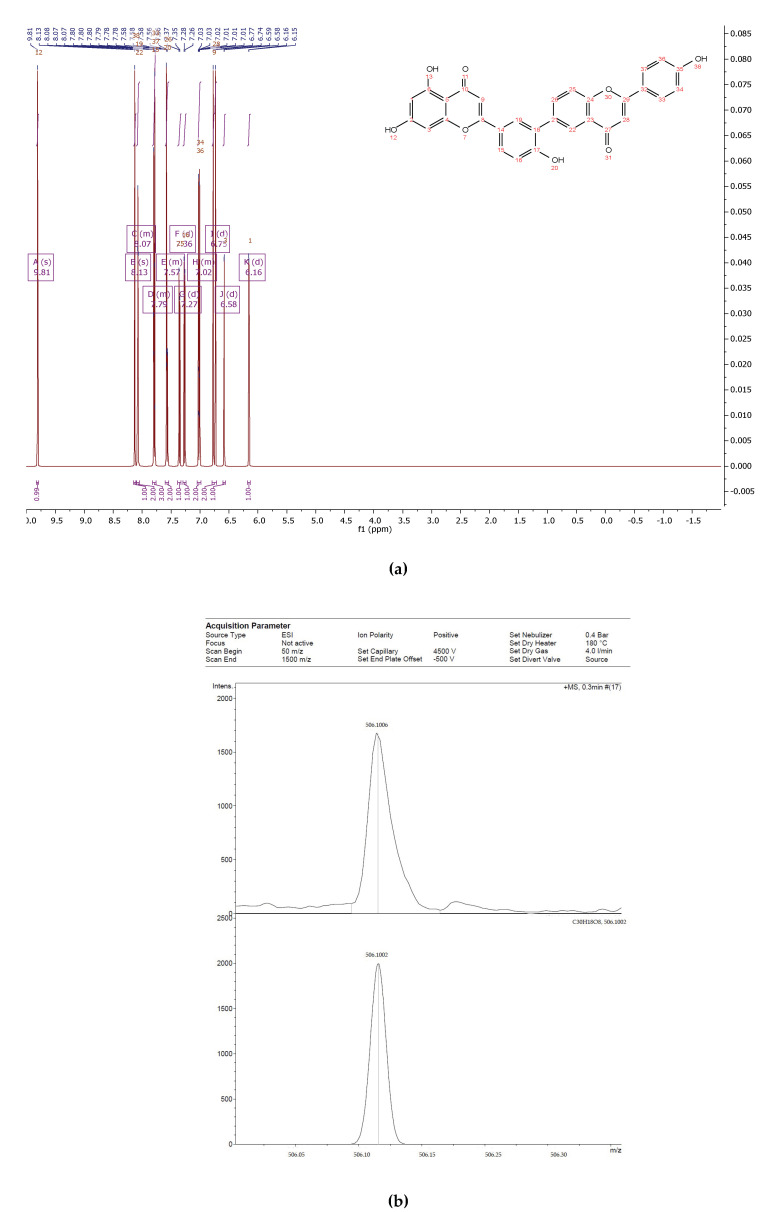
^1^H NMR (**a**) and HRMS (**b**) spectrum of 5,7-dihydroxy-2-(4-hydroxy-3-(2-(4-hydroxyphenyl)-4-oxo-4H-chromen-6-yl)phenyl)-4H-chromen-4-one from extracts of callus, cell suspension, and root cultures of *Rhodiola rosea*.

**Figure 6:**
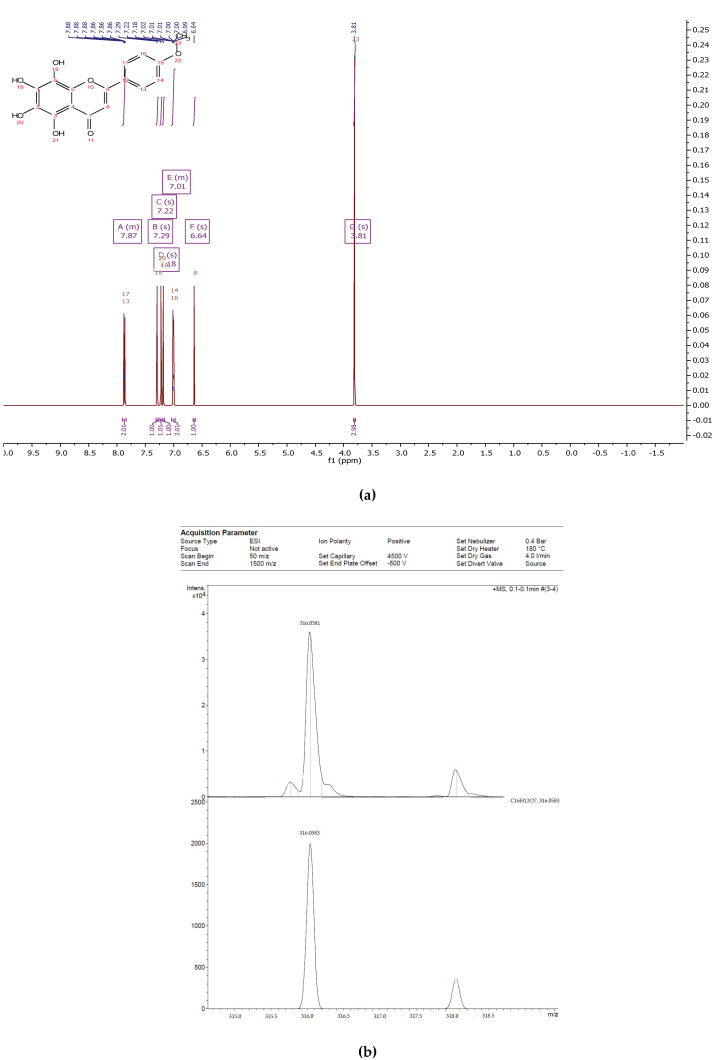
^1^H NMR (**a**) and HRMS (**b**) spectrum of 5,6,7,8-tetrahydroxy-4-methoxyflavone from extracts of callus, cell suspension, and root cultures of *Rhodiola rosea*.

The flavonoid fraction of in vitro callus, cell suspension, and root cultures of *Rhodiola rosea* was separated by preparative HPLC to obtain individual compounds (Figure 1). The structure of individual compounds was determined by ^1^H NMR spectrometry. Five different flavonoids were isolated, and their structure and yield were established (Figure 2, Figure 3, Figure 4, Figure 5 and Figure 6).

**Figure 7:**
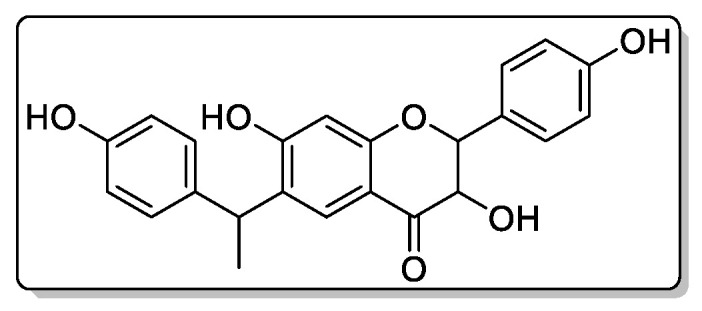
This is chemical formula of the compound **1** (Figure 4).

Compound **1**—6-C-[1-(4-hydroxyphenyl)ethyl]aromadendrin (Figure 7), 25 mg of the compound was isolated: the yield was 0.21%.

^1^H NMR (500 MHz, Chloroform-d) 8.03 (s, 1H), 7.87 (d, *J* = 1.0 Hz, 1H), 7.65 (s, 1H), 7.37–7.30 (m, 3H), 7.15–7.09 (m, 2H), 6.84–6.77 (m, 2H), 6.70–6.64 (m, 2H), 6.57 (s, 1H), 5.14–5.08 (m, 1H), 4.53 (dd, *J* = 7.6, 6.7 Hz, 1H), 4.35–4.23 (m, 2H), 1.42 (d, *J* = 6.2 Hz, 3H). 13C (125 MHz, MeOD) δ 193.11, 160.64, 160.18, 158.28, 156.20, 138.66, 129.74, 128.95, 128.49, 127.91, 126.85, 115.79, 115.40, 114.09, 102.19, 82.91, 73.91, 39.07, 21.72.

HREIMS *m*/*z* 392.1268 (calculated for C23H20O6, 392.1260).

**Figure 8:**
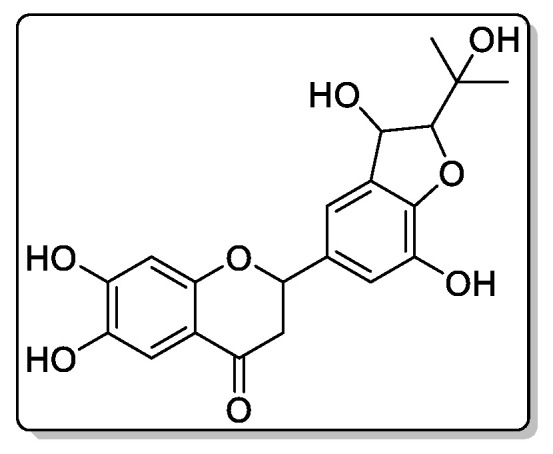
This is chemical formula of the compound **2** (Figure 3).

Compound **2**—2-(3,7-dihydroxy-2-(2-hydroxypropan-2-yl)-2,3-dihydrobenzofuran-5-yl)-6,7-dihydroxychroman-4-one (Figure 8), 23 mg of the compound was isolated: the yield was 0.2 %.

^1^H NMR (500 MHz, Chloroform-d) δ 9.13 (s, 1H), 7.43 (s, 1H), 7.19–7.11 (m, 3H), 7.05 (s, 1H), 6.63 (s, 1H), 5.65 (d, *J* = 6.0 Hz, 1H), 5.45 (tt, *J* = 6.6, 1.1 Hz, 1H), 5.10 (ddd, *J* = 8.0, 6.0, 1.0 Hz, 1H), 4.43 (d, *J* = 7.9 Hz, 1H), 3.46 (s, 1H), 3.08 (dd, *J* = 15.9, 6.4 Hz, 1H), 2.95 (dd, *J* = 16.1, 6.4 Hz, 1H), 1.49 (s, 3H), 1.44 (s, 3H). 13C NMR (125 MHz, Common NMR Solvents) δ 192.79, 155.90, 154.91, 146.76, 141.81, 140.22, 132.65, 126.74, 117.01, 116.94, 112.72, 112.26, 103.83, 96.26, 79.02, 73.46, 71.98, 44.81, 25.31.

HREIMS *m*/*z* 388.1154 (calculated for C20H20O8, 388.1158).

**Figure 9:**
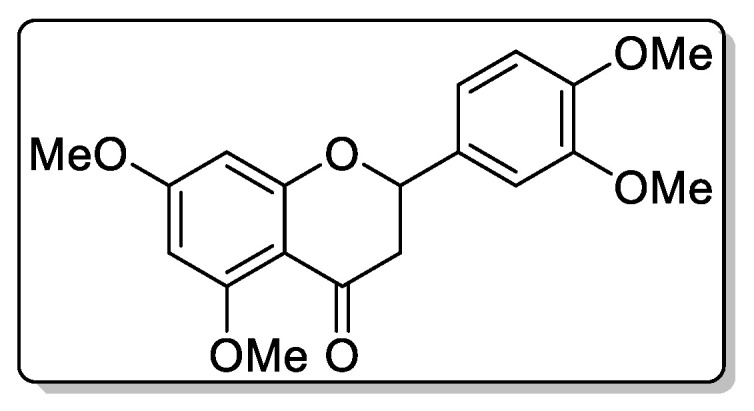
This is chemical formula of the compound **3** (Figure 4).

Compound **3**—2-(3,4-dimethoxyphenyl)-5,7-dimethoxychroman-4-one (Figure 9), 175 mg of the compound was isolated: the yield was 1.5% and the structure identified by NMR spectrometry.

^1^H NMR (500 MHz, Chloroform-d) δ 7.25–7.15 (m, 2H), 6.90–6.84 (m, 2H), 6.28 (d, *J* = 2.2 Hz, 1H), 5.43 (tt, *J* = 6.4, 1.0 Hz, 1H), 3.88–3.80 (m, 12H), 2.80 (dd, *J* = 16.8, 6.5 Hz, 1H), 2.60 (dd, *J* = 16.9, 6.6 Hz, 1H).

HREIMS *m*/*z* 344.1268 (calculated for C19H20O6, 344.1260).

**Figure 10:**
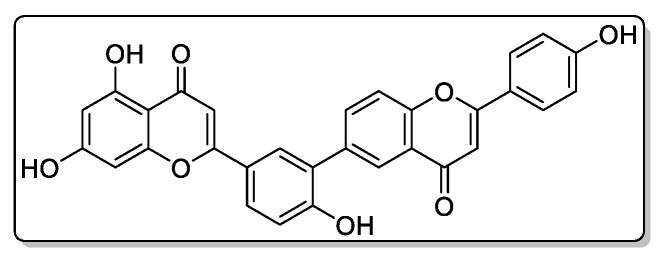
This is chemical formula of the compound **4** (Figure 5).

Compound **4**—5,7-dihydroxy-2-(4-hydroxy-3-(2- (4-hydroxyphenyl)-4-oxo-4H-chromen-6-yl) phenyl)-4H-chromen-4-one (Figure 10), 45 mg of the compound was isolated: the yield was 0.5%.

^1^H NMR (500 MHz, Chloroform-d) δ 9.81 (s, 1H), 8.13 (s, 1H), 8.10–8.05 (m, 2H), 7.82–7.76 (m, 3H), 7.60–7.54 (m, 2H), 7.36 (d, *J* = 8.4 Hz, 1H), 7.27 (d, *J* = 8.4 Hz, 1H), 7.05–6.99 (m, 2H), 6.75 (d, *J* = 18.3 Hz, 2H), 6.58 (d, *J* = 1.8 Hz, 1H), 6.16 (d, *J* = 1.8 Hz, 1H).

HREIMS *m*/*z* 506.1006 (calculated for C30H18O8, 506.1002).

**Figure 11:**
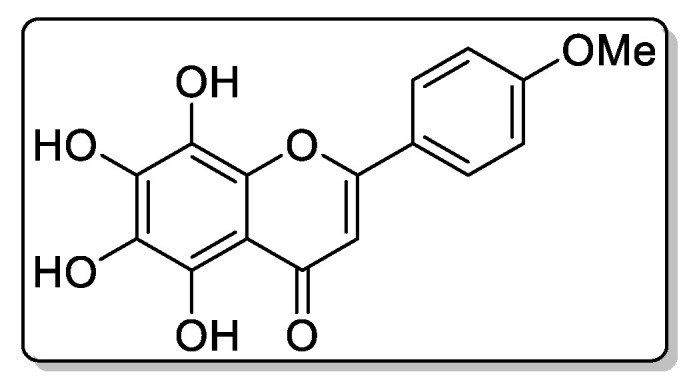
This is chemical formula of the compound **5** (Figure 6).

Compound **5**—5,6,7,8-tetrahydroxy-4-methoxyflavone (Figure 11), 0.35 mg of the compound was isolated: the yield was 0.37%.

^1^H NMR (500 MHz, DMSO-d6): d 7.85 (2H, 11 d, J ¼ 8:5 Hz, H-20 and H 60), 6.92 (2H, d, J ¼ 8:5 Hz, H-30 and H-50, 6.61 (1H, s, H), 3.78 (3H, s, CH3O).

HREIMS *m*/*z* 316.0581 (calculated for C16H12O7, 316.0583).

## 4. Discussion

A fraction of the sum of flavonoids from in vitro callus, suspension cells, and root cultures of *Rhodiola rosea* was isolated by HPLC. The total content of the sum of flavonoids was 2.32% from the sample weight of in vitro callus, cell suspension, and root cultures (Figure 1). The results of the qualitative composition analysis of the dried biomass extracts of in vitro callus, cell suspension, and root cultures showed that the main biologically active substances in the medicinal plant, *Rhodiola rosea*, are 6-C-[1-(4-hydroxyphenyl)ethyl]aromadendrin (25 mg, yield 0.21%), 2-(3,7-dihydroxy-2-(2-hydroxypropan-2-yl)-2,3-dihydrobenzofuran-5-yl)-6,7-dihydroxychroman-4-one (23 mg, yield 0.2%), 2-(3,4-dimethoxyphenyl)-5,7-dimethoxychroman-4-one (175 mg, yield 1.5%), 5,7-dihydroxy-2-(4-hydroxy-3-(2-(4-hydroxyphenyl)-4-oxo-4H-chromen-6-yl)phenyl)-4H-chromen-4-one (45 mg, yield 0.5%), and 5,6,7,8-tetrahydroxy-4-methoxyflavone (0.35 mg, 0.5%).

In Mendoza et al. [26], three new ecdysteroids were discovered: polypodine B 20,22-acetonide, 20-hydroxyecdysone 2,3; 20,22-diacetonide and isovitexiron, together with 20-hydroxyecdysone, 20-hydroxyecdysone 2,3-acetonide, 20-hydroxyecdysone 20,22-acetonide, ajugasterone C, macisterone A and polypodyne B, were isolated from root cultures of *Leuzea carthamoides*. The described data confirm the presence of ecdysteroids in the root cultures of *Rhodiola rosea*, which we found in our study. Identification of the molecular structure for all individual compounds was carried out by NMR spectrometry and high-resolution mass spectrometry (HRMS) analysis. In the results of this study, we proposed a method for the isolation of individual compounds with high yield, selectivity, and identification by NMR spectra, with a high-selectivity-characterized value of chemical shift signal and multiplet structure.

The study by Dyshlyuk et al. [27] describes the BAS profiles of callus, cell suspension, and root cultures of *Thevetia peruviana*, an ornamental shrub growing in many tropical regions of the world. This plant produces BAS with biological properties of interest to the pharmaceutical industry. Extracts were prepared in 50% aqueous ethanol and ethyl acetate. Phytochemical analysis was performed using standard chemical tests and thin-layer chromatography. Additionally, during the growth of callus, cell suspension, and root cultures, the total amount of phenolic and flavonoid compounds (TPC and TFC), the total amount of cardiac glycosides (TCG), and the total antioxidant activity (TAA) were determined. The phenolic chemical profile was also analyzed by high-performance liquid chromatography (HPLC). Common BASs (alkaloids, amino acids, antioxidants, cardiac glycosides, leukoanthocyanidins, flavonoids, phenols, sugars, and triterpenes) were found in all samples. HPLC analysis revealed dihydroquercetin, a flavonoid with anti-cancer properties. These results demonstrate the usefulness of *T. peruviana* callus, cell suspension, and root cultures for the production of valuable pharmaceutical compounds. The data presented in [27,28] confirm the high accumulation of flavonoids and ecdysteroids in vitro callus, cell suspension, and root cultures of medicinal plants.

In [29], ginseng callus, cell suspension, and root cultures, as well as their extracts, were studied. Biologically active substances were extracted with 30 to 70% ethanol. Organic compounds were determined using thin-layer chromatography. Quercetin, magneferin, luteolin, rutin, quercetin-2-D-glucoside, malvidin, as well as caffeic, cinnamic, ferulic, and sinapic acids, were identified for each plant. The described results confirm the high accumulation of flavonoids and ecdysteroids in the extracts of in vitro callus, cell suspension, and root cultures of medicinal plants.

## 5. Conclusions

The qualitative composition analysis of extracts of callus, cell suspension, and root cultures showed that ecdysteroids and flavonoids are the most promising BAS from the perspective of industrial and technological production. These compounds make the greatest contribution to the BAS complex of extracts of callus, cell suspension, and root cultures; their biological activity has been established, and their technological production is cost-effective since it allows these compounds to be sold on the existing market, thereby reducing economic risk. Many people nowadays prefer natural dietary supplements to synthetic medicines. Therefore, in vitro biotechnological production of callus, cell suspension, and root cultures under controlled conditions represents a cost-effective way for the commercial mass-production of phytochemicals. The studied extracts of callus, cell suspension, and root cultures of the medicinal plant, *Rhodiola rosea*, are readily available and are considered effective, with fewer side effects compared to modern drugs in the treatment of various diseases. BASs of this plant are planned for use in the production of pharmaceuticals and dietary supplements with antitumor, antimicrobial, and antioxidant effects. Further research will be focused on the optimization of conditions for growing in vitro callus, cell suspension, and root cultures of medicinal plants for the accelerated synthesis of metabolites.

## Data Availability

Data is available on request.
